# Synthesis and characterisation of (*Z*)-styrylbenzene derivatives as potential selective anticancer agents

**DOI:** 10.1080/14756366.2018.1513925

**Published:** 2018-09-23

**Authors:** Ya-Bing Xin, Jia-Jun Li, Hong-Jian Zhang, Jun Ma, Xin Liu, Guo-Hua Gong, Yu-Shun Tian

**Affiliations:** a Key Laboratory of Natural Resources and Functional Molecules of the Changbai Mountain, Affiliated Ministry of Education, College of Pharmacy, Yanbian University, Yanji, P.R. China;; b Jiangsu Hansoh Pharmaceutical Group Co., Ltd., Lianyungang, P.R. China;; c First Clinical Medical College of Inner Mongolia University for Nationalities, Tongliao, P.R. China;; d Inner Mongolia Key Laboratory of Mongolian Medicine Pharmacology for Cardio-Cerebral Vascular System, Inner Mongolia University for Nationalities, Tongliao, P.R. China

**Keywords:** Styrylbenzene, cyano, selective toxic effect, anticancer, synthesis

## Abstract

To identify anticancer agents with high potency and low toxicity, a series of (*Z*)-styrylbenzene derivatives were synthesised and evaluated for anticancer activities using a panel of nine cancer cell lines and two noncancerous cell lines. Most derivatives exhibited significant anti-proliferative activities against five cancer cell lines, including MGC-803 and BEL-7402. (*Z*)-3-(*p*-Tolyl)-2-(3,4,5-trimethoxyphenyl)acrylonitrile (**6h**) showed a strong inhibitory effect on MGC-803 cells (IC_50_ < 0.01 µM) and exhibited stronger anti-proliferative activity than taxol (IC_50_ < 0.06 ± 0.01 µM). The IC_50_ value of **6h** in L-02 cells was 10,000-fold higher than in MGC-803 cells. Compound **6h** inhibited proliferation of BEL-7402 cells by arresting at the G2/M phase through up-regulation of cyclin B1 expression, down-regulation of cyclin A and D1 expression, and induction of apoptosis. In addition, **6h** inhibited the migration of BEL-7402 cells and the formation of cell colonies.

## Introduction

**Figure 1.**
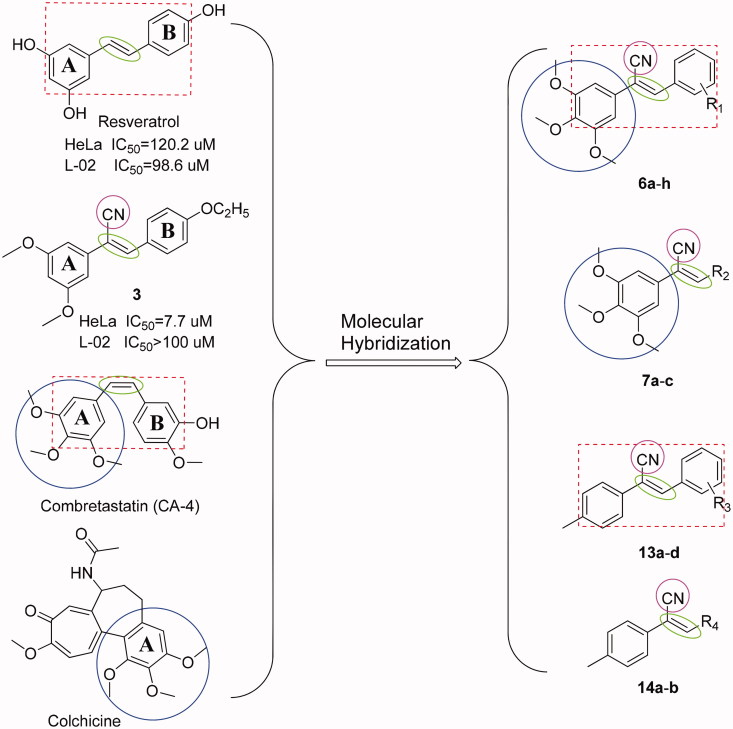
The structures of resveratrol, compound **3**, CA-4, colchicine, and the design of target compounds **6a**-**h**, **7a**-**c**, **13a**-**d**, and **14a**-**b**.

Cancer is becoming an increasingly important disease and is a leading cause of death worldwide^1^. Although a variety of anticancer agents are currently available, none is able to eradicate cancer cells without having toxic effects on healthy tissues^2^. Therefore, finding new anticancer agents with higher potency and lower toxicity is a great challenge^3^^,^^4^. More than 60% of anticancer drugs used in the clinic originate from natural sources^5^. Agents containing a styrylbenzene structure, such as resveratrol ((*E*)-3,5,4′-trihydroxystilbene)^6^ and CA-4 ((*Z*)-2-methoxy-5–(3,4,5-trimethoxystyryl) phenol)^7^, are naturally present in medicinal plants (Figure 1) and have been shown to exert anticancer effects with high toxicity to normal cells^6–8^.

The microtubule system of eukaryotic cells is an important target for the development of anticancer agents^9^. Recently, much attention has been devoted to studying microtubule colchicine-binding sites of antimitotic agents, such as CA-4 and its analogs^10–13^. Compounds with a structure similar to that of colchicine and having trimethoxy groups on the A benzene ring (Figure 1) may have potential as anticancer drugs. For example, it was reported that compounds with *O*-methylation on the A ring of the styrylbenzene structure displayed significant anticancer effects^14^.

Cyano is highly polar, small in size, has electron-withdrawing properties and is able to react with key amino acid residues of active sites on a variety of proteins^15^. In a previous experiment, compound **3** (Figure 1), which had a cyano group on the ethylene bond, was found to have better selective toxic effect against HeLa cells with fewer toxic effects on non-cancerous L-02 cells when compared with the commercially available drug taxol^2^.

On the basis of the previous study, we retained the trimethoxy groups on the A benzene ring, introduced a cyano group into the vinyl bridge to maintain its *Z* isomer, and either changed the substituents on the B benzene ring or replaced the B benzene ring with other heterocyclic structures to design various styrylbenzene derivatives. We also replaced the 3,4,5-trimethoxy groups with *p*-methyl groups to further study the effect of trimethoxy groups on anticancer activity and selective toxic effects (Figure 1).

## Materials and methods

### Chemistry

^1^H NMR and ^13^C NMR spectra were obtained on an AV-300 (Bruker, Switzerland), and using TMS as internal standard. The chemical shifts are given in ppm referenced to the respective solvent peak, and coupling constants (*J*) are reported in hertz (Hz). High-resolution mass spectra were measured on a MALDI-TOF/TOF mass spectrometer (Bruker Dartonik, Germany). IR spectra were recorded (in KBr) on a FTIR1730 instrument. All reagents were obtained commercially and used were of analytical grade. Reaction processes were tracked by TLC on silica gel plates. Melting points of target compounds were determined in the open glass capillaries and were uncorrected.

### *General procedure for synthesis of compounds* 6a-h, 7a-c, 13a-d*, and* 14a-b

3,4,5-Trihydroxybenzoic acid (**1**) and 4-methylbenzoic acid (**8**) were used as starting materials to synthesise 5-(bromomethyl)-1,2,3-trimethoxybenzene (**4**) and (bromomethyl) benzene (**11**), respectively. Next, a mixture of 10 mmol of compound **4** or **11**, 1.485 g (15 mmol) of TMSCN (trimethylsilyl cyanide), 4.725 g (15 mmol) of TBAF (tetrabutyl-ammonium fluoride), and 30 ml of acetonitrile was refluxed for 4–6 h with stirring. The reflux process was monitored by TLC. The reaction mixture was cooled to room temperature (RT), and then was added to 200 ml of ice water with severe stirring. The mixture was filtered, washed with 50% methanol, and purified by recrystallisation from 95% ethanol, yielding a final product of compound **5** or **12**^16–20^.

**Figure 2.**
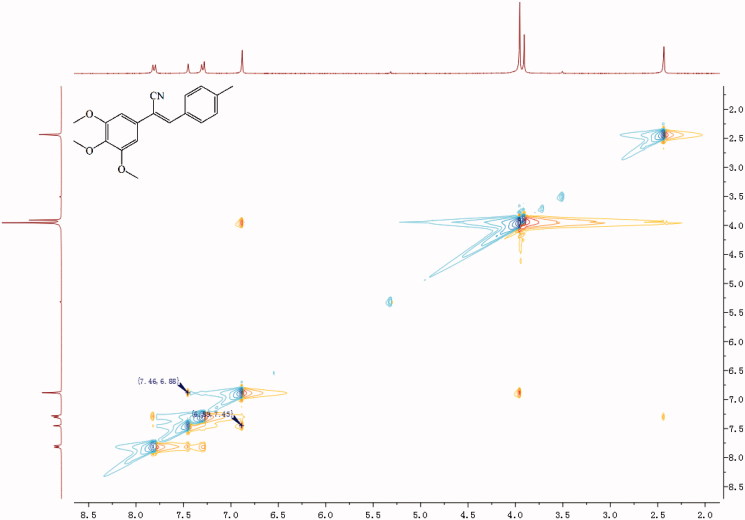
NOE (nuclear overhauser effect) result of compound **6h**.

**Scheme 1.**
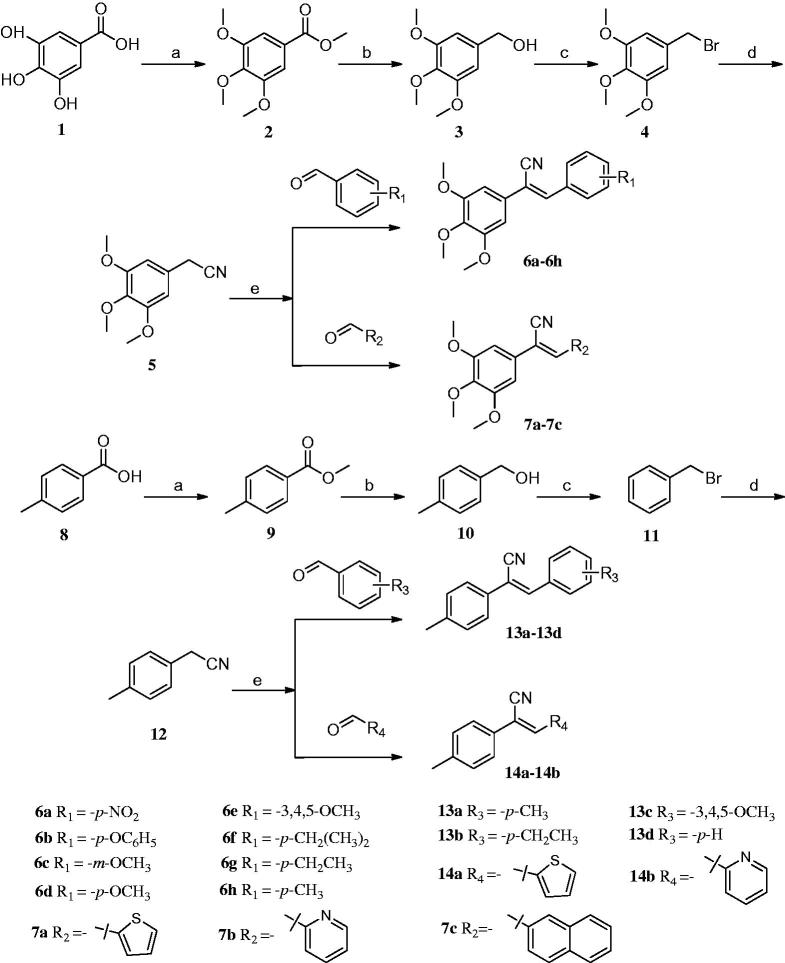
Reagents and conditions: (a) (CH_3_)_2_SO_4_, (CH_3_)_2_CO, K_2_CO_3_, reflux; (b) LiAlH_4_, THF, 0 °C-rt, 4–6 h; (c) CH_2_Cl_2_, PBr_3_, 0 °C-rt, 3–6 h; (d) CH_3_CN, TMSCN, TBAF, reflux, 4–6 h; (e) CH_3_OH, CH_3_ONa, aromatic aldehydes, 4–6 h.

A mixture of 1 mmol compounds **5** or **12**, 1 mmol properly substituted benzaldehyde or aromatic heterocyclic aldehyde, and 10 ml of methanol was heated to 60 °C with stirring. After 30 min, sodium methoxide (0.027 g, 0.5 mmol) was added to the mixture, and the mixture was kept at 60 °C for 4–6 h. The reaction was monitored by TLC. Once complete, the reaction mixture was cooled to RT, and the precipitate was separated by filtration and recrystallised from methanol to give the target final compound (Scheme 1). Because of a cyano-steric effect, the final structure was fixed as the *Z* isomer, confirmed by the nuclear Overhauser effect (NOE) (Figure 2). The detailed information on the structural and physiochemical characteristics of the final target compounds can be found in the Supplementary material.

## Biological evaluation

### Materials

3-(4,5-Dimethylthiazol-2-yl)-2,5-diphenyl-2*H*-tetrazolium bromide (MTT) was purchased from Sigma-Aldrich Co. (St. Louis, MO, USA). The propidium iodide (PI) and Annexin V-FITC apoptosis detection kit were purchased from Invitrogen (Eugene, OR, USA).

### Cell lines and cell culture

All human cell lines were used in this study. MGC-803 late-stage differentiation gastric cancer cell, A549 lung cancer cell, HepG2 hepatoma cell, AGS gastric cancer cell, BEL-7402 liver cancer cell, HCT-116 colorectal cancer cell, HeLa cervical cancer cell, SGC-7901 early differentiation gastric cancer cell, L-02 normal liver cell, MCF-7 breast cancer cell, and MCF-10A normal breast cell were initially purchased from American Type Culture Collection (ATCC, Manassas, VA, USA). RPMI-1640 media, Dulbecco’s modified Eagle’s medium (DMEM), and foetal bovine serum (FBS) were provided from Gibco Company (Grand Island, NY, USA). The cells were maintained in DMEM or RPMI-1640, supplemented with 10% FBS, 100 IU/mL penicillin and 100 mg/mL streptomycin (Grand Island, NY, USA), and at 37 °C in a humidified atmosphere containing 5% CO_2_.

### Cell growth inhibition assay

Cells were plated in 96-well plates at a density of 30–40% to ensure exponential growth throughout the experimental period, and then allowed to adhere for 1 day^21^. Next, the cells were treated with four serial concentrations (1, 10, 50, and 100 µM) of each compound. Taxol, CA-4, and CA-4P (phosphorylated form of CA-4) were used as positive controls. After 48 h incubation, MTT solution was added to each well at a final concentration of 2 mg/mL, and incubated for an additional 4 h. Next, the MTT solution was removed and 150 µL of dimethyl sulphoxide (DMSO)/well was added (Sigma-Aldrich, St Louis, MO, USA). The plates were shaken vigorously at RT to ensure complete solubilisation, and absorption at 492 nm was determined. At least three independent experiments were conducted, and the results were summarised in Table 1.

**Table 1.** *In vitro* anti-proliferative activity of the synthesised compounds, CA-4, CA-4P, and taxol against eleven cell lines^a^ (IC_50_ (µM^b^))

Agent	R	IC_50_ (μM)										
		MGC-803	A549	HepG-2	AGS	Bel-7402	HCT116	L-02	HeLa	SGC-7901	MCF-10A	MCF-7
**6a**	4-NO_2_	>100	>100	>100	>100	>100	>100	>100	>100	>100	>100	>100
**6b**	4-OBn	>100	>100	>100	>100	>100	>100	>100	>100	>100	>100	>100
**6c**	3-OCH_3_	22.6 ± 9.25	>100	>100	>100	>100	>100	>100	>100	>100	>100	65.13 ± 6.64
**6d**	4-OCH_3_	6.18 ± 0.36	>100	>100	0.05 ± 0.003	0.75 ± 0.21	1.51 ± 0.05	4.15 ± 4.16	>100	>100	3.07 ± 0.18	16.66 ± 4.77
**6e**	3.4.5-OCH_3_	>100	>100	>100	44.56 ± 9.11	31.24 ± 2.01	32.1 ± 5.25	70.6 ± 7.65	>100	>100	15.56 ± 2.45	32.88 ± 17.6
**6f**	4-CH_2_(CH_3_)_2_	7.21 ± 3.65	36.62 ± 0.134	>100	1.33 ± 0.15	0.72 ± 0.01	0.17 ± 0.1	>100	>100	>100	5.79 ± 0.48	4.37 ± 2.80
**6g**	4-CH_2_CH_3_	<0.01	>100	>100	4.38 ± 2.04	0.65 ± 0.05	0.34 ± 0.01	>100	>100	>100	1.19 ± 0.08	>100
**6h**	4-CH_3_	<0.01	0.53 ± 0.023	3.74 ± 1.73	0.51 ± 0.45	0.15 ± 0.03	0.61 ± 0.16	>100	>100	>100	9.45 ± 5.25	2.61 ± 0.98
**7a**	Thiophene-2-yl	0.67 ± 0.22	>100	>100	8.01 ± 1.65	1.82 ± 1.20	16.58 ± 10.26	21.2 ± 10.75	>100	>100	55.81 ± 10.4	15.02 ± 2.00
**7b**	Pyridine-2-yl	>100	>100	>100	>100	44.6 ± 32.7	>100	>100	>100	>100	33.44 ± 17.4	15.95 ± 5.67
**7c**	Naphthalene-1-yl	3.45 ± 2.11	>100	>100	3.51 ± 0.25	2.53 ± 0.12	4.58 ± 0.42	8.21 ± 0.051	>100	>100	1.03 ± 0.44	2.83 ± 0.35
**13a**	4-CH_3_	>100	>100	>100	27.86 ± 5.84	>100	>100	>100	>100	>100	>100	>100
**13b**	4-CH_2_CH_3_	>100	>100	>100	26.53 ± 3.89	>100	>100	>100	>100	>100	85.69 ± 3.12	35.03 ± 14.8
**13c**	3.4.5-OCH_3_	>100	>100	>100	66.20 ± 3.65	77.3 ± 0.26	79.2 ± 5.21	>100	>100	>100	>100	33.94 ± 7.30
**13d**	4-H	89.21 ± 3.20	>100	>100	>100	>100	>100	>100	>100	>100	>100	>100
**14a**	Thiophene-2-yl	>100	>100	>100	>100	>100	>100	>100	>100	>100	>100	>100
**14b**	Pyridine-2-yl	>100	>100	>100	>100	>100	>100	>100	>100	>100	>100	>100
**CA-4**		0.06 ± 0.02	>100	>100	3.42 ± 0.03	2.02 ± 0.03	0.16 ± 0.00	1.10 ± 0.03	>100	0.30 ± 0.00	3.23 ± 1.21	0.22 ± 0.09
**CA-4P**		0.03 ± 0.02	>100	>100	4.73 ± 0.22	5.45 ± 2.04	0.59 ± 0.22	2.67 ± 0.61	>100	0.12 ± 0.03	14.55 ± 1.36	0.48 ± 0.25
**Taxol**		0.06 ± 0.01	0.44 ± 0.02	0.94 ± 0.03	0.02 ± 0.01	0.09 ± 0.02	0.03 ± 0.01	>100	12.95 ± 0.47	>100	0.18 ± 0.02	0.08 ± 0.01

a Cytotoxicity as IC_50_ for each cell line, refers to the concentration of compound which reduced by 50% the optical density of treated cells with respect to untreated cells using the MTT assay.

b Data represent the mean values of three independent determinations.

### Analysis for cell cycle by flow cytometry

BEL-7402 cells were plated 6-well plates with 5.0 × 10^5^ cells/well for overnight (O/N) and were synchronised at the G0 phase by serum starvation (0.1% FBS in RPMI-1640) for 24 h. Then the cells were incubated with 0.01, 0.05, 0.1 µM of **6h** and 0.1 μM of taxol, respectively. Vehicle group was treated with 0.1% DMSO. Following 12 h of incubation, the cells were detached with 0.05% trypsin/5 mM EDTA and centrifuged at 1000 rpm for 10 min, and fixed in 70% ethanol at −20 °C for O/N. Then, the cells were subsequently analysed the cell cycle distribution by flow cytometry using a FACSCalibur flow cytometer with Cell Quest software (Becton-Dickinson, Franklin Lakes, NJ, USA), plotting at least 30,000 events per sample^22^. The percentage of cells in the G0/G1, S, and G2/M phases of the cell cycle were determined using the ModFit LT version 4.0 software package (Verity Software, Topsham, ME, USA).

### Analysis of apoptosis by flow cytometry

Apoptosis was detected using an apoptosis detection kit. BEL-7402 cells were plated in 6-well plates (5.0 × 10^5^ cells/well) and incubated at 37 °C overnight. Exponentially growing cells were then incubated with **6h** at either 0.01 or 0.1 µM, or taxol at 0.1 µM. Vehicle group was treated with 0.1% DMSO. Following 12 h of incubation, cells were analysed for apoptosis as previously described^23^, using a FACSCalibur flow cytometer with Cell Quest software (Becton-Dickinson, Franklin Lakes, NJ, USA).

### Cell migration assay

Quantitative cell migration was performed using 24-well Boyden chambers (Corning, NY, USA) as described^7^. Briefly, transwells with 8-µm pore size filters were inserted into 24-well plates. RPMI-1640 (500 µL) containing 10% FBS was added to the lower chamber, and 100 µL of a serum-free cell suspension (1 × 10^5^ cells) was placed in the upper chamber. The BEL-7402 cells were pre-treated with indicated concentrations of **6h**, taxol, or DMSO. The plates were incubated at 37 °C with 5% CO_2_ for 24 h, and the cells on the upside were removed using cotton swaps. After several rinses, the cells adhering in the lower layer of insert were fixed with methanol and stained with 1× crystal violet solution. The number of migrated cells in nine randomly selected fields from triplicate chambers was counted in each experiment under a phase-contrast microscope at 200× magnification.

### Colony formation assay

A previously described colony formation assay was used^7^ with slight modification. 1 × 10^4^ BEL-7402 cells/well were seeded into 6-well plates and then stimulated with the indicated concentration of **6h**, taxol, or DMSO. The plates were removed from incubation when colonies were large enough to count (>50 cells). Colonies were then fixed and stained with 1 × Giemsa solution, rinsed, allowed to dry, and counted. All the experiments were repeated independently at least three times. The LD_50_ value for **6h** was determined using SigmaPlot II.

### Western blotting

BEL-7402 cells were harvested in logarithmic growth phase and the adherent and floating cells were collected after 12 h of drug treatment. The collected cells were resuspended in cold RIPA buffer (100 mM NaCl, 100 mM Tris (pH7.4), 10% Triton X-100, 1 mM PMSF, pH7.5) with 0.1 µM leupetin protease inhibitor as well as 1 µg/mL pepstatin and cracked on ice for 30 min. Then according to the reference to did immunoblotting assay^22^.

### Molecular modelling

The molecular docking study was performed using Discovery Studio 2017 Client. In this study, the structure of tubulin (PDB code: 1SA0) downloaded from protein database was chosen for docking. The 3D structures of compounds were provided using Chem 3D, the pdbqt format files of ligand, and the autodock format of protein were provided using AutoDockTools version 1.5.6.

## Results and discussion

### Chemistry

As shown in Scheme 1, through series of reactions, such as esterification, reduction, bromination, substitution, and *Knoevenagel* condensation, two series of derivatives were synthesised^16–20^. In brief, 3,4,5-trihydroxybenzoic acid (**1**) or *p*-methylbenzoic acid (**8**) as the starting material, dimethyl sulphate in the presence of acetone as solvent, potassium carbonate as a catalyst, compound **2** or **9** was obtained. Then, the compound was reduced respectively under ice-cooling with lithium aluminium hydride as a reducing agent, anhydrous tetrahydrofuran as solvent to give compound **3** or **10**. The resulting compound **3** or **10** was dissolved in anhydrous CH_2_Cl_2_ and replaced by PBr_3_ under ice bath to give compound **4** or **11**. Compound **5** or **12** was prepared by dissolving compound **4** or **11** in acetonitrile, with TMSCN and TBAF as catalysts. The series of target compounds were prepared by substituting aromatic aldehydes with compound **5** or **12** in the presence of methanol as solvent and sodium methoxide as catalyst. Before biological evaluation, the compounds were characterised via IR, ^1^H NMR, and 13C NMR spectrometry as well as high-resolution mass spectrometry, and the *Z* isomer was confirmed by NOE.

## Biological evaluation

### *In vitro* cytotoxicity assay and structure-activity relationship (SAR) studies

According to standard protocol^2^, preliminary *in vitro* anti-proliferative activities of the synthesised compounds were evaluated by MTT assay, using nine human cancer cell lines (MGC-803, A549, HepG2, AGS, BEL-7402, HCT116, HeLa, SGC-7901, and MCF-7) and two normal human cell lines (L-02 and MCF-10A), and compared with those of CA-4, CA-4P, and taxol.

Results of the screening are summarised in Table 1. Most compounds showed good anti-proliferative activities against cancer cells including MGC-803, AGS, BEL-7402, HCT-116, and MCF-7. Three alkyl-substituted compounds (**6f**, **6g**, and **6h**), *p*-methoxy-substituted **6d**, thiophene-2-yl derivative **7a**, and naphthalene-1-yl derivative **7c**, all with 3,4,5-trimethoxy groups on the A benzene ring, had lower IC_50_ values (<0.01–7.21 µM) in MGC-803. The order of anticancer activity was *p*-CH_3_ ≈ *p*-CH_2_CH_3_ > thiophene-2-yl > naphthalene-1-yl > *p*-OCH_3_ > *p*-(CH_2_)_2_CH_3_. Compounds **6g** and **6h** exhibited particularly strong inhibitory activities, with IC_50_ values less than 0.01 µM, making them more active than all control agents tested. Similarly, **6d**, **6f**, **6g**, **6h**, **7a**, and **7c** exhibited better anticancer activity (IC_50_ = 0.05, 1.33, 4.38, 0.51, 8.01, 3.51 µM, respectively) in AGS cells. In this assay, **6d** exhibited activity similar to the positive control drug taxol (IC_50_ = 0.02 µM), and stronger activity than CA-4 (IC_50_ = 3.42 µM) and CA-4P (IC_50_ = 4.73 µM). Compounds **6f**, **6g**, **6h**, and **7c** also showed stronger activity than CA-4 and CA-4P against AGS cells. The order of anticancer activity was *p*-OCH_3_ > *p*-CH_3_ > *p*-(CH_2_)_2_CH_3_ > naphthalene-1-yl > *p*-CH_2_CH_3_ > thiophene-2-yl. Analysis using human hepatocellular carcinoma cells (BEL-7402) revealed that compound **6h** had the best anticancer activity (IC_50_ = 0.15 µM), much stronger than CA-4 (IC_50_ = 2.02 µM) and CA-4P (IC_50_ = 5.45 µM). The high anticancer activity of **6h** was followed by compounds **6g**, **6f**, **6d**, **7a**, and **7c** with IC_50_ values of 0.65, 0.72, 0.75, 1.82, and 2.53 µM, respectively. The order of anticancer activity was *p*-CH_3_ > *p*-CH_2_CH_3_ > *p*-(CH_2_)_2_CH_3_ > *p*-OCH_3_ > thiophene-2-yl > naphthalene-1-yl. Compounds **6f**, **6g**, and **6h** exhibited better anticancer activities (IC_50_ = 0.17, 0.34, and 0.61 µM, respectively) than CA-4P (IC_50_ = 0.59 µM) on HCT116 cells. Compounds **6f**, **6h**, and **7c** expressed strong anticancer activity against MCF-7 (human breast adenocarcinoma) cells. Remarkably, compound **6h** was 6-fold more active than taxol against MGC-803 cells and exhibited similar activity to taxol against A549 and BEL-7402 cells. Compound **6h** was superior to CA-4 and CA-4P against almost all cancer cell lines tested, including MGC-803, A549, HepG-2, AGS, and BEL-7402 cells. However, 17 of the synthesised compounds displayed poor anticancer activity against HeLa and SGC-7901 cancer cell lines. In addition, among the compounds having 3,4,5-trimethoxy groups on the A benzene ring, *p*-NO_2_, *p*-OBn, *m*-OCH_3_, and pyridine-2-yl substituted compounds (**6a**, **6b**, **6c**, and **7b**) expressed extremely poor *in vitro* anticancer activities against all nine cancer cell lines, and 3,4,5-trimethoxy substituted compound **6e**, which has a heavy steric effect, also showed weak anticancer activity against all cancer cell lines tested. Synthesised compounds having *p*-methyl groups on the A benzene ring (**13a**–**d** and **14a**–**b**) also demonstrated poor anticancer activities against all cell lines tested.

Using these preliminary results, we identified the following SARs: (1) the anticancer activity of a structure with a 3,4,5-trimethoxy group on the A benzene ring was greater than that of a structure with a *p*-methyl group on the A benzene ring; and (2) in structures containing 3,4,5-trimethoxy groups on the A benzene ring, introducing a B benzene ring with small size *para*-electron-donating groups, such as *p*-CH_3,_*p*-OCH_3_, and *p*-CH_2_CH_3_, or exchanging B benzene with an electron-rich aromatic heterocyclic structure, such as thiophene, significantly improves anticancer activity. Based on these observations, we expect that in structures containing 3,4,5-trimethoxy groups on the A benzene ring, electron density and steric hindrance effects on the B benzene ring strongly influence the anticancer activity of the (*Z*)-styrylbenzene derivatives bearing a cyano group synthesised.

### Selective inhibition of cancer cell growth

Lack of selective cytotoxicity is the main factor restricting the tolerated dose of most conventional chemotherapeutic agents^2^. To address this, we compared the toxicity of all synthesised compounds with CA-4, CA-4P, and taxol on normal human cell lines L-02 and MCF-10A. The calculated selectivity index (SI) of each compound is depicted in Table 2. Compounds **6g** and **6h** exhibited 10,000-fold higher selectivity for MGC-803 cells than for normal L-02 cells, which is a much higher selectivity than displayed by taxol. In addition, many compounds expressed significantly higher selectivity for cancer cells than for L-02 cells, including **6d** for AGS, **6f** and **6g** for BEL-7402 and HCT116, **6h** for A549, AGS, BEL-7402, and HCT116 cells. However, none of the compounds showed strong selectivity for MCF-7 (breast cancer) cells over MCF-10A (normal breast) cells. The control compounds CA-4 and CA-4P displayed poor selectivity for cancer cells over normal cells although they induced strong cytotoxicity in cancer cells. On the basis of these results, we speculated that **6h** was the best candidate for further mechanism analysis. The strong toxicity of **6h** for MGC-803 cells (IC_50_ < 0.01 µM) is likely to introduce experimental error even with small changes in concentration; therefore, BEL-7402 cells, for which **6h** demonstrated moderate toxicity and selectivity, were selected for further study of **6h**.

**Table 2.** Selectivity index values of compounds relative to the effects on normal cell L-02 or MCF-10A cells.

Agent	R	SI						
		IC_50_ (L-02/MGC-803)	IC_50_ (L-02/A549)	IC_50_ (L-02/HepG-2)	IC_50_ (L-02/AGS)	IC_50_ (L-02/BEL-7402)	IC_50_ (L-02/HCT116)	IC_50_ (MCF-10A/MCF-7)
**6d**	4-OCH_3_	–	–	–	>83.0	>5.5	>2.7	–
**6f**	4-CH_2_(CH_3_)_2_	>13.8	>2.7	–	>75.1	>138.8	>588.2	>1.3
**6g**	4-CH_2_CH_3_	>10000	–	–	>22.8	>153.8	>294.1	–
**6h**	4-CH_3_	>10000	>188.6	>26.7	>196.0	>666.6	>163.9	>3.6
**7a**	Thiophene-2-yl	>31.6	–	–	>2.6	>11.6	>1.2	>3.7
**CA-4**		>18.3	–	–	–	–	>6.8	>14.6
**CA-4P**		>89.0	–	–	–	–	>4.5	>30.3
**Taxol**		>1666	>227.2	>106.3	>5000	>1111	>3333	>2.2

–: not suitable for calculation; SI: selectivity index, IC_50_ (L-02 or MCF-10A)/IC_50_ (cancer cell).

### *Cell cycle regulation by compound* 6h *and Western blot analysis*

**Figure 3.**
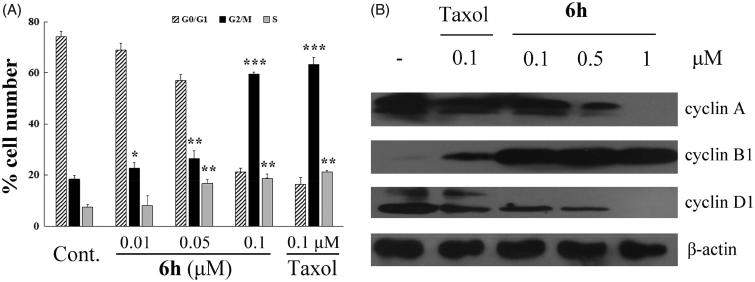
Compound **6h** decreases the expression of cyclin A and cyclin D1 and increases the expression of cyclin B1, thus reducing the cell cycle progression of BEL-7402 cells. (A) Cell numbers in the phases of the cell cycle were analyzed on 12 h after stimulation. Values were obtained from three separate experiments and represent the mean ± SD. *p < 0.05. **p < 0.01. ***p < 0.001 compared to respective control group. (B) Immunoblots were probed using antibodies directed against cyclin A, B1, and D1, and β-actin. Whole-cell protein lysates were extracted from BEL-7402 cells cultured after 12 h. Results are representative of three separate experiments.

The cell-cycle is a series of events that result in cell division, duplication, and proliferation. Numerous cytotoxic compounds exert their anti-proliferative effect by inducing cell-cycle arrest, apoptosis, or both^2^. These mechanisms are considered effective anticancer strategies^21^. To study the mechanism by which compound **6h** reduced the viability of BEL-7402 cells, we used flow cytometry to analyse cell-cycle distribution. As shown in Figure 3(A), 6h increased the percentage of G2/M cell population in a concentration-dependent manner from 23.09% to 59.99% after 12 h incubation, and the trend was similar in the S cell population (4.44–19.35%). After treatment with 0.1 µM **6h**, the cell distribution at G0/G1, G2/M, and S phases was very similar to the 0.1 µM taxol treatment group. This finding suggests that **6h** induces cell-cycle arrest at the S and G2/M phases.

Since entry into the S phase in the cell-cycle requires the accumulation of cell-cycle activation-related cyclins^7^, we assessed the expression levels of cyclins, such as cyclin A and cyclin D1 to determine whether the decreased cell proliferation by **6h** involved these proteins. The **6h** treatment group had lower expression of cyclin A and D1 compared with the vehicle group, this reduced expression was dose-dependent, and similar to that of the taxol treatment group at 0.1 µM (Figure 3(B)). Cyclin B1 plays an important role in the transition from interphase to mitotic phase and governs cell cycle progression by enhancing cell-cycle distribution in the G2/M fraction^24^, and it has been reported that expression of cyclin B1 is increased by exposure to anticancer agents^25^. In our experiment, **6h** treatment induced increased expression of cyclin B1 compared with treatment with vehicle or 0.1 µM of taxol. These results suggest that the down-regulation of cyclins A and D1 and up-regulation of cyclin B1 are related to the decrease in cell proliferation induced by **6h** treatment on BEL-7402 cells.

### *Apoptotic effects by compound* 6h

**Figure 4.**
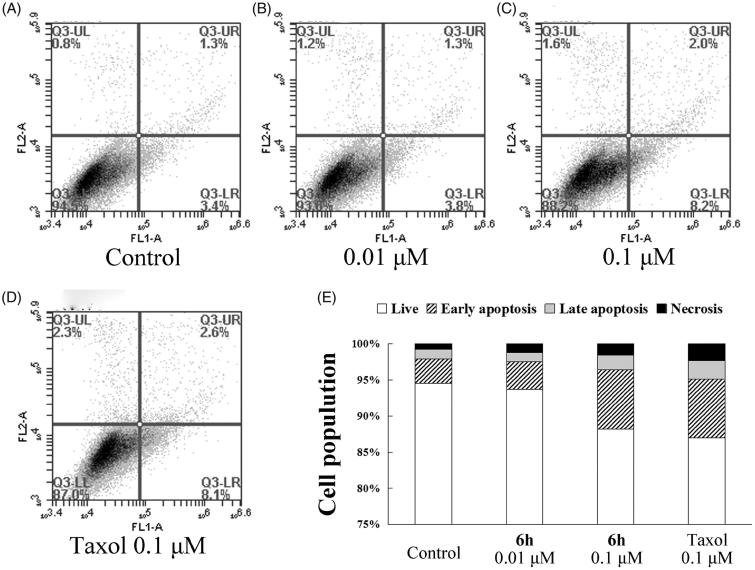
Apoptosis induction in BEL-7402 cells after treatment for 12 h with (A) 0.1% DMSO (vehicle); (B) 0.01 μM **6h**; (C) 0.1 μM **6h**; (D) 0.1 μM taxol. (E) The results of the cell apoptosis.

To further investigate whether **6h** induces apoptosis, BEL-7402 cells were treated with vehicle, **6h** (0.01 or 0.1 µM), or taxol (0.1 µM) for 12 h, then stained with Annexin V-FITC and PI. As shown in Figure 4, the percentage of total apoptotic cells (early + late apoptotic cells) increased in a dose-dependent manner with **6h** treatment, but the effect was weak. Treatment with 0.1 µM **6h** resulted in a similar number of total apoptotic cells to the taxol treatment group (10.2% and 10.7%, respectively), both of which were higher than the vehicle treatment group (4.7%). These results indicate that apoptosis plays only a slightly important role in the inhibition of BEL-7402 cell proliferation by **6h**.

### *Cell migration and colony formation inhibition by compound* 6h

**Figure 5.**
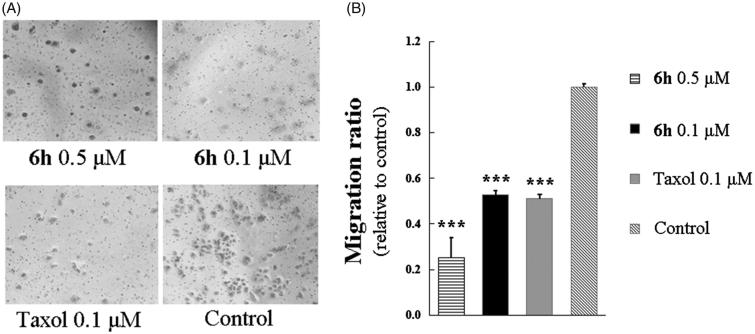
Transwell assay of compound **6h** showing inhibition of BEL-7402 cell migration. (A) The images of stained BEL-7402 cells adhering in the lower layer of insert of transwell with phase-contrast microscopy (200× magnification). (B) The relative migration ratio of BEL-7402 cells. Data expressed as mean ± SD (*n* ≥ 3). ***p < 0.001 compared to control group.

Aggressive tumours have a strong ability to proliferate and migrate, and in many cases, cell mobility affects cell proliferation^2^. We used a transwell migration assay to investigate the effect of compound **6h** on the migration of BEL-7402 cells. As shown in Figure 5, treatment with 0.1 or 0.5 µM of **6h** resulted in migration ratios that were markedly decreased compared with the control group. A similar finding was observed for taxol at 0.1 µM.

**Figure 6.**
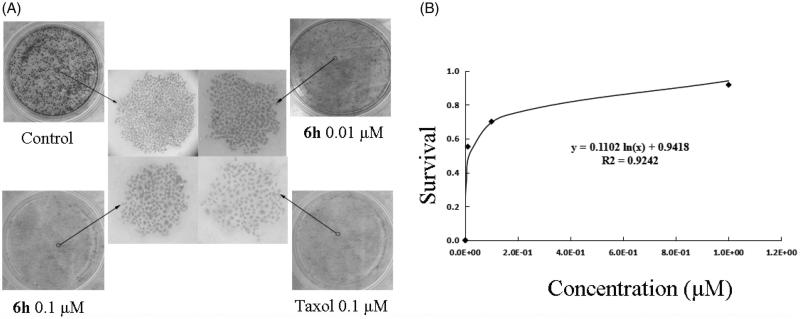
Colony formation ability was inhibited by **6h** treatment. (A) The images of stained colonies under phase-contrast microscopy. (B) The curve was fitted as the inhibition ratio of colony formation against the concentration. Values are represented as mean ± SD from three independent colony formation experiments.

The LD_50_ of **6h** was determined using a colony formation assay with BEL-7402 cells over a relatively long exposure time (> 10 days) to **6h**. As shown in Figure 6, **6h** had a dose-dependent inhibitory effect on colony formation, with an LD_50_ value of 0.056 ± 0.0047 µM, further confirming the anticancer activity of the compound. Compared with the dense and large cell colonies of control group, the **6h** treatment groups showed sparse and small cell colonies which was observed in a dose-dependent manner. Trypan blue staining was used to further investigate the cytotoxicity of **6h**. Treatment of cells with **6h** at concentrations ranging from 0.001 µM to 10 µM resulted in percentages of dead cells that were negligible (data not shown). These results indicate that **6h** has a strong anti-proliferative effect on BEL-7402 cells in the absence of evident cytotoxicity.

### Molecular docking

**Figure 7.**
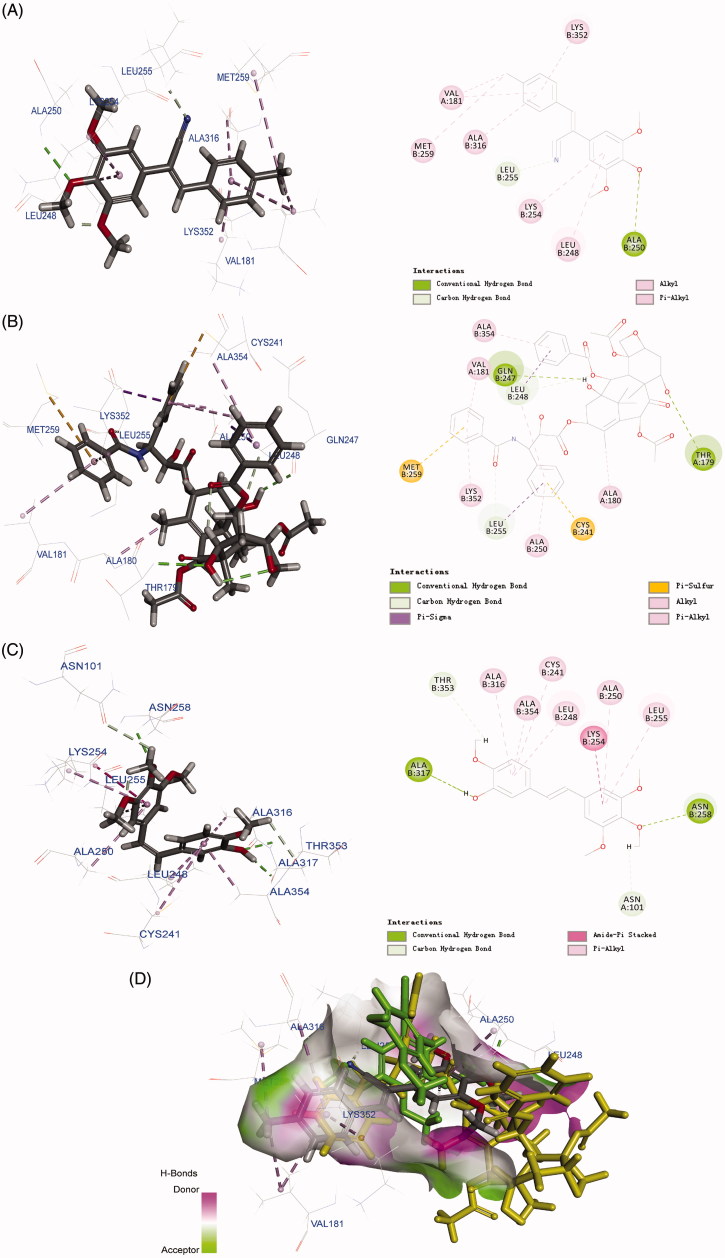
Molecular modelling of compounds in complex with tubulin (PDB: 1SA0). Shown is the proposed binding mode and interaction between tubulin and selected compounds. (A) **6h**, (B) taxol, and (C) CA-4. The compounds and important amino acids in the binding pockets are shown in stick model. (D) Superposition of **6h**, taxol, and CA-4 in the pocket of tubulin in surface representation. The compounds are shown in stick model with carbon atoms in grey (**6h**), yellow-green (taxol), or green (CA-4).

To investigate the possible binding modes of the compound with tubulin, **6h**, a well-behaved and selectively toxic compound, was used in molecular docking studies. The structure of tubulin used in the docking study was obtained from the Protein Data Bank (PDB code: 1SA0, https://www.rcsb.org/structure/1SA0)^26^. All docked conformations are ranked based on docking scores. Taxol and CA-4 were used as positive controls. As shown in Figure 7 (A–C), **6h**, taxol, and CA-4 interacted with tubulin residues differently. Compound **6h** formed abundant chemical bonds with the amino acid residues on the β chain of tubulin and showed strong affinity, whereas the number of chemical bonds formed with the amino acid residues on the α chain was sparse and showed poor affinity. The cyano group tended to bind to the β subunit but not the α subunit. In the case of the *cis* isomer of CA-4, the entire structure made multiple contacts with the β subunit. The 2D diagram effectively displays these structural features, highlighting several binding regions on the β subunit, including Cys241, Leu248, Ala316, and Ala354. Taxol bound between the α and β subunits of tubulin, the three benzene rings docked into the β subunit side while the rest of the compound docked into the α subunit. The 2D image shows several binding sites on the α subunit, including Val181, but most of the binding contacts are on the β subunit. The superposition of the three compounds in the biding pocket clearly shows the binding posture of **6h** and taxol are similar, whereas the biding location of CA-4 is completely different from them (Figure 7(D)). On the basis of the molecular modelling results, we suggest that **6h** selectively inhibits cancer cell proliferation without toxicity to normal cell proliferation by targeting both the α and β subunits of tubulin.

## Conclusions

A series of (*Z*)-styrylbenzene derivatives, containing a cyano group, were designed, synthesised, and evaluated for anti-proliferative properties against nine human cancer cell lines and two non-cancerous human cell lines. Most derivatives exhibited significant anticancer activity against five of the cancer cell lines, including MGC-803 and BEL-7402 cells. SAR analysis indicated that in structures containing 3,4,5-trimethoxy groups on the A benzene ring, the electron density and steric effect of the B benzene ring strongly affect the anticancer activity of the derivatives. Most compounds, especially **6h**, exhibited notably high potency against a set of cancer cell lines. Compound **6h** possessed the highest anticancer activity against MGC-803 cells, being 6-fold more potent than taxol and CA-4. The IC_50_ value of **6h** in L-02 cells was more than 10,000-fold higher than in MGC-803 cells and more than 666-fold higher than in BEL-7402 cells, indicating that **6h** exhibits selective toxic effects. In addition, **6h** arrested BEL-7402 cells in the G2/M phase of the cell cycle and slightly induced apoptosis. Mechanistic studies suggested that the blockade of G2/M phase was associated with up-regulation of cyclin B1 and down-regulation of cyclins A and D1. Molecular modelling results suggested the selectivity of **6h** to inhibit only cancer cell proliferation was achieved by targeting both the α and β subunits of tubulin. Compound **6h** inhibited the migration of BEL-7402 cells and the formation of cell colonies. In summary, these newly developed compounds showed marked biological activity *in vitro* and have potential for further development as a novel class of selective anticancer agents.

## Supplementary Material

Supplemental_material.doc
