# Supplementary material for: Synthesis and characterisation of (Z)-styrylbenzene derivatives as potential selective anticancer agents
Source: J Enzyme Inhib Med Chem. 2018 Sep 23;33(1):1554–64. doi: 10.1080/14756366.2018.1513925 (PMC6161602; doi:10.1080/14756366.2018.1513925)
Supplement: Supplemental_material.doc [file IENZ_A_1513925_SM5087.doc]

**Synthesis and characterisation of (*Z*)-styrylbenzene derivatives as potential selective anticancer agents**

Ya-Bing Xin^a#^, Jia-Jun Li^a#^, Hong-Jian Zhang^a^, Jun Ma^b^, Xin Liu^a^, Guo-Hua Gong^c,d^* & Yu-Shun Tian^a^*

*^a^Key Laboratory of Natural Resources and Functional Molecules of the Changbai Mountain, Affiliated Ministry of Education, College of Pharmacy, Yanbian University, Yanji, Jilin Province 133002, PR China*

*^b^Jiangsu Hansoh Pharmaceutical Group Co., Ltd. lianyungang, jiangsu Province 222000, PR China*

*^c^First Clinical Medical College of Inner Mongolia University for Nationalities, Tongliao, Inner Mongolia 028002, PR China*

*^d^Inner Mongolia Key Laboratory of Mongolian Medicine Pharmacology for Cardio-Cerebral Vascular System, Inner Mongolia University for Nationalities, No. 22 Holin He Street, Tongliao, Inner Mongolia 028002, PR China*

Data of IR, NMR, and HRMS spectra of the compounds

***(*Z*)-3-(4-Nitrophenyl)-2-(3,4,5-trimethoxyphenyl)acrylonitrile (6a)***

Yellow powder, mp 174-176°C, yield 45%. IR (KBr) cm^-1^: 1355.72 (NO_2_ s), 1450.22, 1469.50 (C=C arom.), 1523.50 (NO_2_ as), 2215.83 (C≡N), 3085.75 (H–C=). ^1^H NMR (300 MHz, CDCl_3_): *δ* 8.21 (d, *J*=8.3Hz, 2H, C11, C13-H), 8.06 (s, 2H, C10, C14-H), 7.52 (s, 1H, C8-H), 6.90 (s, 2H, C4, C6-H), 3.93 (d, *J* = 12.8 Hz, 6H, C1, C3-OCH_3_), 3.91 (s, 3H, C2-OCH_3_). ^13^C NMR (75 MHz, CDCl_3_): *δ* 153.60 (s, C12), 148.39 (s, C1, C3), 138.19 (s, C8), 135.16 (s, C9), 133.76 (s, C2), 130.02 (s, C10, C14), 128.86 (s, C5), 124.42 (d, *J* = 10.1 Hz, C11, C13), 117.07 (s, C7-CN), 114.94 (s, C7), 103.49 (s, C4, C5, C6), 60.94 (s, C2-OCH_3_), 56.27 (s, C1, C3-OCH_3_). HRMS calcd. for C_18_H_16_N_2_O_5_ ([M＋H]^＋^): 340.1089; found: 340.1203.

1. ***-3-(4-(Benzyloxy)phenyl)-2-(3,4,5-trimethoxyphenyl)acrylonitrile (6b)***

Light yellow powder, mp 92-94°C, yield 35%. IR (KBr) cm^-1^: 1018.24, 1253.51 (CAr–O), 1432.86, 1465.65 (C=C arom.), 1600.64 (C=C), 2211.97 (C≡N), 3043.15 (H–C=). ^1^H NMR (300 MHz, CDCl_3_): *δ* 7.88 (d, *J* = 8.7 Hz, 2H, C10, C14-H), 7.52 – 7.37 (m, 6H, C8-H, C12-OPh), 7.06 (d, *J* = 8.4 Hz, 2H, C11, C13-H), 6.84 (s, 2H, C4, C6-H), 3.93 (s, 6H, C1, C3-OCH_3_), 3.89 (s, 3H, C2-OCH_3_). ^13^C NMR (75 MHz, CDCl_3_): *δ* 160.50 (s, C12), 153.51 (s, C15), 141.31 (s, C1, C3), 131.09 (s, C8), 130.72 – 130.36 (m, C2), 128.65 (s, C10, C14), 128.18 (s, C17, C19), 127.45 (s, C9), 126.57 (s, C5), 125.83 (s, C18), 118.45 (d, *J* = 6.8 Hz, C7-CN), 115.23 (s, C11, C13), 108.71 (s, C16, C20), 103.16 (s, C7), 90.34 (s, C4, C6), 60.96 (s, C2-OCH_3_), 56.27 (s, C1, C3-OCH_3_). HRMS calcd. for C_24_H_21_NO_4_ ([M＋H]^＋^): 387.1471; found: 387.3159.

**(Z)-*3-(3-Methoxyphenyl)-*2-(3,4,5-trimethoxyphenyl)acrylonitrile (6c)**

Light yellow powder, mp 144-146°C, yield 60%. IR (KBr) cm^-1^: 1029.81, 1255.44 (C_Ar_–O), 1488.79, 1511.93 (C=C arom.), 1600.64 (C=C), 2213.90 (C≡N), 3058.57 (H–C=). ^1^H NMR (300 MHz, CDCl_3_): *δ* 7.48 (s, 1H, C8-H), 7.44 (s, 1H, C13-H), 7.41 (t, *J* = 1.7 Hz, 1H, C14-H), 7.38 (s, 1H, C10-H), 7.00 (d, *J* = 7.9 Hz, 1H, C12-H), 6.87 (s, 2H, C4, C6-H), 3.94 (s, 6H, C1, C3-OCH_3_), 3.89 (s, 3H, C11-OCH_3_), 3.88 (s, 3H, C2-OCH_3_). ^13^C NMR (75 MHz, CDCl_3_): *δ* 153.54 (s, C11), 153.22 (s, C1, C3), 141.48 (s, C8), 140.32 (s, C2), 139.24 (s, C9), 130.08 (s, C13), 128.83 (s, C5), 118.23 (s, C14), 110.33 (s, C7-CN), 106.74 (s, C10, C12), 103.55 (s, C7), 96.23 (s, C4, C6), 60.85 (s, C2-OCH_3_), 56.24 (d, *J* = 6.7 Hz, C1, C3, C11-OCH_3_). HRMS calcd. for C_19_H_19_NO_4_ ([M＋H]^＋^): 325.1314; found: 325.2715.

***(*Z*)-3-(4-Methoxyphenyl)-2-(3,4,5-trimethoxyphenyl)acrylonitrile (6d)***

White powder, mp 156-158°C, yield 44%. IR (KBr) cm^-1^: 1243.87, 1251.59 (CAr–O), 1456.72, 1506.15 (C=C arom.), 2206.10 (C≡N), 3025.29 (H–C=). ^1^H NMR (300 MHz, CDCl_3_): *δ* 7.88 (d, *J* = 8.8 Hz, 2H, C10, C14-H), 7.39 (s, 1H, C8-H), 7.03 – 6.94 (m, 2H, C11, C13-H), 6.84 (s, 2H, C4, C6-H), 3.93 (s, 6H, C1, C3-OCH_3_), 3.88 (s, 3H, C2-OCH_3_), 3.88 (s, 3H, C12-OCH_3_). ^13^C NMR (75 MHz, CDCl_3_): δ 161.32 (s, C12), 153.50 (s, C1, C3), 141.24 (s, C8), 138.94 (s, C2), 130.99 (s, C10, C14), 130.43 (s, C9), 126.35 (s, C5), 118.40 (s, C7-CN), 114.31 (s, C11, C13), 108.61 (s, C7), 103.35 (s, C4, C6), 60.83 (s, C2-OCH_3_), 56.26 (s, C1, C3-OCH_3_), 55.31 (s, C12-OCH_3_). HRMS calcd. for C_19_H_19_NO_4_ ([M＋H]^＋^): 325.1314; found: 325.3101.

***(*Z*)-2,3-Bis(3,4,5-trimethoxyphenyl)acrylonitrile (6e)***

White powder, mp 84-86°C, yield 55%. IR (KBr) cm^-1^: 1081.88, 1253.51 (CAr–O), 2208.11 (C≡N), 3081.72 (H–C=). ^1^H NMR (300 MHz, CDCl_3_): *δ* 7.36 (s, 1H, C8-H), 7.18 (s, 2H, C10, C14-H), 6.85 (s, 2H, C4, C6-H), 3.94 (d, *J* = 1.7 Hz, 12H, C1, C3, C11, C13-OCH_3_), 3.91 (d, *J* = 10.4 Hz, 6H, C2, C12-OCH_3_). ^13^C NMR (75 MHz, CDCl_3_): *δ* 153.50 (s, C1, C3), 153.18 (s, C11, C13), 141.58 (s, C8), 140.10 (s, C12), 139.00 (s, C2), 130.11 (s, C9), 128.86 (s, C5), 118.30 (s, C7-CN), 110.30 (s, C7), 106.55 (s, C10, C14), 103.29 (s, C4, C6), 60.93 (s, C2, C12-OCH_3_), 56.21 (d, *J* = 5.7Hz, C1, C3, C11, C13-OCH_3_). HRMS calcd. for C_21_H_23_NO_6_ ([M＋H]^＋^): 385.4104; found: 385.1523.

***(*Z*)-3-(4-Isopropylphenyl)-2-(3,4,5-trimethoxyphenyl)acrylonitrile (6f)***

Light gray powder, mp 156-158°C, yield 59%. IR (KBr) cm^-1^: 1064.52, 1251.59 (C_Ar_–O), 1423.22, 1511.93 (C=C arom.), 2211.97 (C≡N), 3067.94 (H–C=). ^1^H NMR (300 MHz, CDCl_3_): *δ* 7.83 (d, *J* = 8.3 Hz, 2H, C10, C14-H), 7.44 (s, 1H, C8-H), 7.33 (d, *J* = 8.2 Hz, 2H, C11, C13-H), 6.86 (s, 2H, C4, C6-H), 3.94 (s, 6H, C1, C3-OCH_3_), 3.88 (d, *J* = 2.8 Hz, 3H, C2-OCH_3_), 2.98 (dd, *J* = 13.8, 7.0 Hz, 1H, C12-CH(CH_3_)_2_), 1.28 (d, *J* = 6.9 Hz, 6H, C12-CH(CH_3_)_2_). ^13^C NMR (75 MHz, CDCl_3_): *δ* 153.51 (s, C1, C3), 151.88 (s, C12), 141.73 (s, C8), 138.96 (s, C2), 131.23 (s, C9), 130.30 (s, C10, C14), 129.34 (s, C11, C13), 127.03 (s, C5), 118.23 (s, C7-CN), 110.34 (s, C7), 103.31 (s, C4, C6), 60.94 (s, C2-OCH_3_), 56.27 (s, C1,C3-OCH_3_), 34.10 (s, C12-CH(CH)_3_), 23.67 (s, C12-CH(CH)_3_). HRMS calcd. for C_21_H_23_NO_3_ ([M＋H]^＋^): 337.4122; found: 337.1676.

***(*Z*)-3-(4-Ethylphenyl)-2-(3,4,5-trimethoxyphenyl)acrylonitrile (6g)***

Light yellow powder, mp 76-78°C, yield 63%. IR (KBr) cm^-1^: 1064.52, 1247.73 (CAr–O), 1463.72, 1501.36 (C=C arom.), 2204.26 (C≡N), 3162.15 (H–C=). ^1^H NMR (300 MHz, CDCl_3_): *δ* 7.82 (d, *J* = 8.2 Hz, 2H, C10, C14-H), 7.44 (s, 1H, C8-H), 7.31 (d, *J* = 8.1 Hz, 2H, C11, C13-H), 6.87 (s, 2H, C4, C6-H), 3.94 (s, 6H, C1, C3-OCH_3_), 3.89 (s, 3H, C2-OCH_3_), 2.71 (q, *J* = 7.6 Hz, 2H, C12-CH_2_CH_3_), 1.27 (t, *J* = 7.6 Hz, 3H, C12-CH_2_CH_3_). ^13^C NMR (75 MHz, CDCl_3_): *δ* 153.51 (s, C1, C3), 147.31 (s, C12), 141.76 (s, C8), 138.95 (s, C2), 131.11 (s, C9), 130.31 (s, C10, C14), 129.31 (s, C11, C13), 128.46 (s, C5), 118.22 (s, C7-CN), 110.35 (s, C7), 103.30 (s, C4, C6), 60.95 (s, C2-OCH_3_), 56.28 (s, C1,C3-OCH_3_), 28.83 (s, C12-CH_2_CH_3_), 15.20 (s, C12-CH_2_CH_3_). HRMS calcd. for C_20_H_21_NO_3_ ([M＋H]^＋^): 323.3856; found: 323.1519.

***(*Z*)-3-(p-Tolyl)-2-(3,4,5-trimethoxyphenyl)acrylonitrile (6h)***

White acicular crystal, mp 168-170°C, yield 35%. IR (KBr) cm^-1^: 1000.06 1243.87 (CAr–O), 1463.72, 1506.15 (C=C arom.), 2206.10 (C≡N), 3062.15 (H–C=). ^1^H NMR (300 MHz, CDCl_3_): *δ* 7.79 (d, *J* = 8.1 Hz, 2H, C10, C14-H), 7.43 (s, 1H, C8-H), 7.29 (s, 2H, C17, C19-H), 6.86 (s, 2H, C4, C6-H), 3.92 (d, *J* = 11.2 Hz, 6H, C1, C3-OCH_3_), 3.89 (s, 3H, C2-OCH_3_), 2.42 (s, 3H, C12-CH_3_). ^13^C NMR (75 MHz, CDCl_3_): *δ* 153.50 (s, C1, C3), 141.74 (s, C8), 141.04 (s, C12), 130.87 (s, C2), 130.28 (s, C9), 129.62 (s, C10, C14), 129.18 (s, C11, C13), 128.06 (s, C5), 118.19 (s, C7-CN), 110.32 (s, C7), 103.28 (s, C4, C6), 60.93 (s, C2-OCH_3_), 56.26 (s, C1, C3-OCH_3_), 21.51 (s, C12-CH_3_). HRMS calcd. for C_19_H_19_NO_3_ ([M＋H]^＋^): 309.3591; found: 309.1363.

***(*Z*)-3-(Thiophen-2-yl)-2-(3,4,5-trimethoxyphenyl)acrylonitrile (7a)***

Light yellow powder, mp 102-104°C, yield 58%. IR (KBr) cm^-1^: 1448.29 (C=C arom heter.), 2206.18 (C≡N), 3087.50 (H–C=). ^1^H NMR (300 MHz, CDCl_3_): *δ* 7.67 (d, *J* = 3.7 Hz, 1H, C12-H), 7.58 (s, 1H, C8-H,), 7.55 (d, *J* = 5.0 Hz, 1H, C10-H), 7.19 – 7.12 (m, 1H, C11-H), 6.83 (s, 2H, C4, C6-H), 3.94 (s, 6H, C1, C3-OCH_3_), 3.88 (s, 3H, C2-OCH_3_). ^13^C NMR (75 MHz, CDCl_3_): *δ* 153.51 (s, C1, C3), 138.85 (s, C8), 137.73 (s, C2), 133.62 (s, C10), 132.18 (s, C9), 129.86 (s, C12), 129.46 (s, C11), 127.78 (s, C5), 118.12 (s, C7-CN), 108.08 (s, C7), 102.98 (s, C4, C6), 60.91 (s, C2-OCH_3_), 56.22 (s, C1, C3-OCH_3_). HRMS calcd. for C_16_H_15_NO_3_S ([M＋H]^＋^): 301.0773; found: 301.3527.

***(*Z*)-3-(Pyridin-2-yl)-2-(3,4,5-trimethoxyphenyl)acrylonitrile (7b)***

Light brown powder, mp 128-130°C, yield 48%. IR (KBr) cm^-1^: 1429.01, 1550.21 (C=C arom heter.), 2210.90 (C≡N), 3031.58 (H–C=). ^1^H NMR (300 MHz, CDCl_3_): *δ* 8.75 (d, *J* = 4.1 Hz, 1H, C13-H), 8.02 (d, *J* = 8.0 Hz, 1H, C10-H), 7.81 (t, *J* = 7.8 Hz, 1H, C11-H), 7.60 (s, 1H, C12-H), 7.39 – 7.27 (m, 1H, C8-H), 6.95 (s, 2H, C4, C6-H), 3.93 (s, 6H, C1, C3-OCH_3_), 3.89 (s, 3H, C2-OCH_3_). ^13^C NMR (75 MHz, CDCl_3_): *δ* 153.49 (s, C1, C3), 152.06 (s, C9), 149.92 (s, C13), 140.39 (s, C8), 136.75 (s, C11), 129.37 (s, C2), 124.13 (s, C5), 123.77 (s, C10, C12), 117.31 (s, C7-CN), 114.61 (s, C7), 103.60 (s, C4, C6), 60.91 (s, C2-OCH_3_), 56.24 (s, C1, C3-OCH_3_). HRMS calcd. for C_17_H_16_N_2_O_3_ ([M＋H]^＋^): 296.1161; found: 296.46137.

***(*Z*)-3-(Naphthalen-1-yl)-2-(3,4,5-trimethoxyphenyl)acrylonitrile (7c)***

Light yellow powder, mp 118-120°C, yield 41%. IR (KBr) cm^-1^: 1419.36, 1583.20 (C=C arom.), 1600.64 (C=C), 2213.09 (C≡N), 3058.57 (H–C=). ^1^H NMR (300 MHz, CDCl_3_): *δ* 8.20 (s, 1H, C14-H), 8.05 (d, *J* = 7.2 Hz, 1H, C8-H), 7.95 (td, *J* = 9.5, 4.0 Hz, 3H, C13, C15, C18-H), 7.59 (s, 3H, C16, C17, C12-H), 6.96 (s, 2H, C4, C6-H), 3.96 (s, 6H, C1, C3-OCH_3_), 3.92 (s, 3H, C2-OCH_3_). ^13^C NMR (75 MHz, CDCl_3_): *δ* 153.64 (s, C1, C3), 139.80 (s, C8), 133.49 (s, C2), 131.48 (s, C11), 131.11 (s, C10), 130.71 (s, C9), 129.78 (s, C12), 128.95 (s, C14), 126.98 (d, *J* = 7.3 Hz, C18), 126.42 (s, C17), 125.52 (s, C15), 123.34 (s, C13), 117.70 (s, C-5), 117.31 (s, C16),115.24 (s, C7-CN), 114.61 (s, C7),103.62 (s, C4, C6), 61.00 (s, C2-OCH_3_), 56.35 (s, C1, C3-OCH_3_). HRMS calcd. for C_22_H_19_NO_3_ ([M＋H]^＋^): 345.1365; found: 345.2657.

***(*Z*)-2,3-Di-p-tolylacrylonitrile (13a)***

White powder, mp 68-70°C, yield 80%. IR (KBr) cm^-1^: 1465.65, 1587.35 (C=C arom.), 2215.83 (C≡N), 3025.79 (H–C=). ^1^H NMR (300 MHz, CDCl_3_): *δ* 7.79 (d, *J* = 8.2 Hz, 2H, C10, C14-H), 7.56 (d, *J* = 8.3 Hz, 2H, C11, C13-H), 7.47 (s, 1H, C8-H), 7.28 (d, *J* = 3.3 Hz, 2H, C4, C6-H), 7.23 (s, 2H, C1, C3-H), 2.39 (t, *J* = 8.5 Hz, 6H, C1, C12-CH_3_). ^13^C NMR (75 MHz, CDCl_3_): *δ* 141.20 (s, C8), 140.79 (s, C12), 139.07 (s, C2), 131.77 (s, C9), 131.09 (s, C5), 129.63 (s, C10, C14), 129.56 (s, C11, C13), 129.14 (s, C1, C3), 125.69 (s, C4, C6), 118.26 (s, C7-CN), 110.31 (s, C7), 21.47 (s, C12-CH_3_), 21.13 (s, C2-CH_3_). HRMS calcd. for C_17_H_15_N ([M＋H]^＋^): 233.1204; found: 233.4357.

***(*Z*)-3-(4-Ethylphenyl)-2-(p-tolyl)acrylonitrile (13b)***

White schistic crystal, mp 112-114°C, yield 58%. IR (KBr) cm^-1^: 1469.50, 1587.14 (C=C arom.), 2215.83 (C≡N), 3085.57 (H–C=). ^1^H NMR (300 MHz, CDCl_3_): *δ* 7.82 (d, *J* = 8.2 Hz, 2H, C10, C14-H), 7.59 – 7.54 (m, 2H, C11, C13-H), 7.48 (s, 1H, C8-H), 7.29 (d, *J* = 8.3 Hz, 2H, C4, C6-H), 7.23 (s, 2H, C1, C3-H), 2.70 (d, *J* = 7.6 Hz, 2H, C13-CH_2_CH_3_), 2.40 (s, 3H, C1-CH_3_), 1.27 (t, *J* = 7.6 Hz, 3H, C13-CH_2_CH_3_). ^13^C NMR (75 MHz, CDCl_3_): *δ* 147.04 (s, C12), 141.19 (s, C8), 139.05 (s, C2), 131.75 (s, C9), 131.28 (s, C5), 129.62 (s, C10, C14), 129.23 (s, C1, C3), 128.36 (s, C11, C13), 125.68 (s, C4, C6), 118.27 (s, C7-CN), 110.31 (s, C7), 28.78 (s, C12-CH_2_CH_3_), 21.13 (s, C2-CH_3_), 15.20 (s, C12-CH_2_CH_3_). HRMS calcd. for C_18_H_17_N ([M＋H]^＋^): 247.1361; found: 247.3106.

***(*Z*)-2-(p-Tolyl)-3-(3,4,5-trimethoxyphenyl)acrylonitrile (13c)***

White powder, mp 135-137°C, yield 47%. IR (KBr) cm^-1^: 1072.24 1205.21 (CAr–O), 1484.93, 1500.26 (C=C arom.), 2221.61 (C≡N), 2962.15 (H–C=). ^1^H NMR (300 MHz, CDCl_3_): *δ* 7.61 – 7.50 (m, 2H, C4, C6-H), 7.41 (s, 1H, C8-H), 7.25 (d, *J* = 4.9 Hz, 2H, C1, C3-H), 7.15 (dd, *J* = 13.7, 4.7 Hz, 2H, C11, C15-H), 3.92 (t, *J* = 4.1 Hz, 9H, C12, C13, C14-OCH_3_), 2.39 (d, *J* = 4.2 Hz, 3H, C2-CH_3_). ^13^C NMR (75 MHz, CDCl_3_): *δ* 153.20 (s, C11, C13), 141.05 (s, C8), 139.21 (s, C12), 131.64 (s, C2), 129.68 (s, C5), 129.41 (d, *J* = 40.3 Hz, C1, C3, C9), 125.71 (s, C4, C6), 118.37 (s, C7-CN), 110.37 (s, C7), 106.54 (s, C10, C14), 60.94 (s, C12-OCH_3_), 56.19 (s, C11, C13-OCH_3_), 21.14 (s, C2-CH_3_). HRMS calcd. for C_19_H_19_NO_3_ ([M＋H]^＋^): 309.3591; found: 309.1364.

***(*Z*)-3-Phenyl-2-(p-tolyl)acrylonitrile (13d)***

White powder, mp 145-147°C, yield 58%. IR (KBr) cm^-1^: 1419.36, 1556.29 (C=C arom.), 2215.83 (C≡N), 3056.65 (H–C=). ^1^H NMR (300 MHz, CDCl_3_): *δ* 7.87 (d, *J* = 8.9 Hz, 2H, C10, C14-H), 7.55 (d, *J* = 8.3 Hz, 2H, C11, C13-H), 7.43 (s, 1H, C8-H), 7.28 (s, 1H, C12-H), 7.24 (d, *J* = 8.2 Hz, 2H, C4, C6-H), 6.98 (d, *J* = 8.9 Hz, 2H, C1, C3-H), 3.87 (s, 3H, C6-CH_3_). ^13^C NMR (75 MHz, CDCl_3_): *δ* 141.05 (s, C8), 139.29 (s, C2), 133.82 (s, C9), 131.62 (s, C5), 130.19 (s, C10, C14), 129.64 (s, C12), 129.10 (s, C1, C3), 128.94 (d, *J* = 21.4 Hz, C11, C13), 125.78 (s, C4, C6), 117.95 (s, C7-CN), 111.63 (s, C7), 21.08 (s, C2-CH_3_). HRMS calcd. for C_16_H_13_N ([M＋H]^＋^): 219.1048; found: 219.3506.

***(*Z*)-3-(Thiophen-2-yl)-2-(p-tolyl)acrylonitrile (14a)***

Light yellow powder, mp 128-130°C, yield 58%. IR (KBr) cm^-1^: 1448.29, 1573.75 (C=C arom heter.), 2206.18 (C≡N), 3087.50 (H–C=). ^1^H NMR (300 MHz, CDCl_3_): *δ* 7.64 (dd, *J* = 3.8, 2.9 Hz, 2H, C4, C6-H), 7.55 (d, *J* = 3.1 Hz, 1H, C12-H), 7.54 – 7.50 (m, 2H, C1, C3-H), 7.25 (s, 1H, C8-H), 7.22 (s, 1H, C10-H), 7.15 (dd, *J* = 5.1, 3.8 Hz, 1H, C11-H), 2.39 (s, 3H, C2-CH_3_). ^13^C NMR (75 MHz, CDCl_3_): *δ* 139.07 (s, C2), 138.03 (s, C8), 133.05 (s, C10), 131.78 (s, C9), 131.04 (s, C5), 129.58 (d, *J* = 14.0 Hz, C1, C3), 127.69 (s, C4, C6, C12), 125.49 (s, C11), 118.09 (s, C7-CN), 108.35 (s, C7), 21.07 (s, C2-CH_3_). HRMS calcd. for C_14_H_11_NS ([M＋H]^＋^): 225.0612; found: 225.4371.

***(*Z*)-3-(Pyridin-2-yl)-2-(p-tolyl)acrylonitrile (14b)***

Brown powder, mp 102-104°C, yield 50%. IR (KBr) cm^-1^: 1439.01, 1520.35 (C=C arom heter.), 2205.45 (C≡N), 3021.83 (H–C=). ^1^H NMR (300 MHz, CDCl_3_): *δ* 8.76 (d, *J* = 0.9 Hz, 1H, C13-H), 7.94 (s, 1H, C10-H), 7.80 (s, 1H, C11-H), 7.66 (s, 1H, C12-H), 7.63 (d, *J* = 2.7 Hz, 2H, C4, C6-H), 7.35 – 7.30 (m, 1H, C8-H), 7.28 (t, *J* = 2.7 Hz, 2H, C1, C3-H), 2.40 (s, 3H, C2-CH_3_). ^13^C NMR (75 MHz, CDCl_3_): *δ* 152.24 (s, C9), 149.86 (s, C13), 140.00 (s, C8)139.93 (s, C11), 136.65 (s, C2), 131.04 (s, C5), 129.71 (s, C1, C3), 126.10 (s, C4, C6), 123.87 (s, C10), 123.98 (s, C12),117.42 (s, C7-CN), 114.68 (s, C7), 21.18 (s, C2-CH_3_). HRMS calcd. for C_15_H_12_N_2_ ([M＋H]^＋^): 220.2692; found: 225.6392.
